# The Influence of Maternal Inflammatory Status on Fetal Telomere Length at Birth

**DOI:** 10.3390/biomedicines13081974

**Published:** 2025-08-14

**Authors:** Mircea Diaconu, Flavius Olaru, Ahmed Abu-Awwad, Simona-Alina Abu-Awwad, Tiberiu Dragomir, Geanina Sacarin, Nicolae Ciprian Pilut, Bogdan Sorop, Melisa Bicu, Razvan Nitu

**Affiliations:** 1 Department of Obstetrics and Gynecology, “Victor Babes” University of Medicine and Pharmacy, 300041 Timisoara, Romania; diaconu.mircea@umft.ro (M.D.); olaru.flavius@umft.ro (F.O.); alina.abuawwad@umft.ro (S.-A.A.-A.); bogdan.sorop@umft.ro (B.S.); nitu.dumitru@umft.ro (R.N.); 2 Clinic of Obstetrics and Gynecology, “Pius Brinzeu” County Clinical Emergency Hospital, 300723 Timisoara, Romania; 3 Department XV, Discipline of Orthopedics, Traumatology, “Victor Babes” University of Medicine and Pharmacy, 300041 Timisoara, Romania; ahm.abuawwad@umft.ro; 4 Research Center University Professor Doctor Teodor Șora, “Victor Babes” University of Medicine and Pharmacy, 300041 Timisoara, Romania; 5 Medical Semiology II Discipline, Internal Medicine Department, “Victor Babes” University of Medicine and Pharmacy, Eftimie Murgu Square No. 2, 300041 Timisoara, Romania; 6 Doctoral School, “Victor Babes” University of Medicine and Pharmacy, 300041 Timisoara, Romania; geanina.sacarin@umft.ro (G.S.); melissa.bicu@umft.ro (M.B.); 7 Department of Microbiology, “Victor Babes” University of Medicine and Pharmacy Timisoara, 300041 Timisoara, Romania

**Keywords:** fetal telomere length, maternal inflammation, IL-6/IL-10 ratio, hsCRP, pregnancy biomarkers, biological aging, cord blood DNA, oxidative stress, telomerase regulation

## Abstract

**Background/Objectives**: Fetal telomere length (FTL) at birth is considered a key marker of early biological aging and future disease risk. While chronic inflammation is known to accelerate telomere attrition in adults, limited evidence exists on how maternal inflammation during pregnancy impacts FTL. This study aimed to investigate the association between maternal systemic inflammatory status in late pregnancy and FTL at birth. **Methods**: We conducted a prospective cohort study including 150 clinically healthy pregnant women recruited in the third trimester. Participants were stratified post hoc into an inflammation group (n = 67) and a control group (n = 83) based on circulating inflammatory markers: high-sensitivity C-reactive protein (hsCRP), interleukin-6 (IL-6), TNF-α, and IL-10. Umbilical cord blood was collected at birth, and telomere length was quantified using real-time PCR. Correlation and multivariable linear regression analyses were performed to evaluate associations between maternal inflammation and FTL. **Results**: Mothers in the inflammation group had significantly elevated hsCRP, IL-6, and TNF-α levels, and lower IL-10 concentrations. FTL was significantly shorter in this group compared to the controls. Unlike previous investigations that relied on single pro-inflammatory markers, our study tests a composite immune-balance index (IL-6/IL-10 ratio) together with hsCRP in a prospectively followed cohort of clinically healthy pregnancies. Using its correlation coefficient, the IL-6/IL-10 ratio alone explained approximately 28% of the total variance in fetal telomere length—almost double the variance captured by IL-6 assessed in isolation. IL-6 and hsCRP emerged as independent negative predictors of FTL in multivariable models (β = −0.37 and −0.29, respectively). The IL-6/IL-10 ratio showed the strongest inverse correlation with FTL (r = −0.53, *p* < 0.001). **Conclusions**: Subclinical systemic inflammation in late pregnancy is independently associated with shorter fetal telomere length at birth, highlighting maternal immune imbalance (especially IL-6/IL-10 ratio) as a modifiable determinant of early biological aging. These findings underscore the need to consider maternal inflammatory profiling in pregnancy as a potential target for early-life preventive strategies.

## 1. Introduction

Over the past few decades, the intrauterine environment has become increasingly recognized as a key determinant of lifelong health [1]. The Developmental Origins of Health and Disease (DOHaD) paradigm has brought renewed attention to how maternal physiology can shape fetal biology in ways that extend far beyond birth [2]. Among the various mechanisms proposed to mediate this developmental programming, telomere biology has emerged as a particularly compelling marker of early-life cellular aging [3].

Telomeres, the protective caps at the ends of chromosomes, progressively shorten with each cell division [4]. Their length at birth is now believed to set the pace for an individual’s “biological clock,” influencing aging trajectories and the risk of chronic diseases such as cardiovascular disease, diabetes, and certain cancers [5,6]. Therefore, understanding what influences fetal telomere length (FTL) is crucial for uncovering early-life contributors to later health [7].

One of the most consistent themes in telomere research is the role of chronic inflammation in accelerating telomere attrition [8]. In adult populations, elevated levels of inflammatory markers such as C-reactive protein (CRP), interleukin-6 (IL-6), and tumor necrosis factor-alpha (TNF-α) have been associated with shorter telomeres [9,10]. However, relatively little is known about whether maternal inflammation during pregnancy, even at subclinical levels, can influence the telomere length of the developing fetus.

Pregnancy is characterized by a unique immune modulation, balancing tolerance toward the fetus with readiness to respond to pathogens. A recent review highlights dynamic maternal immune adaptations, such as increased regulatory T cell and uterine NK cell activity alongside preserved innate immune functions (complement activation, pathogen sensing), which together maintain fetal tolerance while ensuring protection against infections [11]. However, when this balance is disrupted by obesity [12], infection, psychosocial stress [13], or metabolic conditions [14], maternal systemic inflammation may rise, potentially impacting fetal development. Emerging evidence suggests that such prenatal inflammatory exposure may not only affect organ development but may also leave lasting epigenetic and genomic marks, including those related to telomere biology [15,16,17].

Recent studies have begun to link higher maternal CRP and IL-6 levels with shorter telomere length in newborns, typically measured from umbilical cord blood [18].

Building on this evidence, the prospective investigation by Lazarides et al. [15] showed that elevated maternal IL-6 and TNF-α are associated with shorter newborn leukocyte telomere length; however, that study quantified only pro-inflammatory cytokines and did not account for compensatory anti-inflammatory mediators such as IL-10 or composite immune-balance metrics. In addition, their sample included pregnancies with psychosocial stress and metabolic comorbidities and collected blood across a broad gestational window (24–40 weeks), limiting temporal specificity. By focusing on clinically uncomplicated pregnancies, standardizing biomarker collection to 34–36 weeks, and modeling both IL-10 and the IL-6/IL-10 ratio, our study addresses these limitations and tests the hypothesis that immune imbalance, not absolute pro-inflammatory load, is the critical driver of fetal telomere attrition.

These findings support the hypothesis that the fetal genome is already susceptible to environmental influences during gestation, particularly through oxidative stress and immune activation pathways.

Despite these promising leads, a significant gap exists in understanding whether—and to what extent—maternal inflammatory status affects telomere dynamics at birth, especially in healthy pregnancies without overt infection or illness. Moreover, most existing studies focus narrowly on a single marker or rely on retrospective data. A more integrated assessment of the maternal inflammatory profile and its relationship with fetal telomere length could enhance our understanding of early-life determinants of aging and disease risk.

Therefore, the present study aims to examine the temporal association between maternal systemic inflammation in late pregnancy and fetal telomere length measured at birth. By quantifying circulating inflammatory markers and correlating them with telomere length in umbilical cord blood, this study seeks to clarify whether subclinical maternal inflammation is an early determinant of fetal biological aging. Ultimately, this line of inquiry could help illuminate novel pathways for preventive strategies during pregnancy, aimed at improving long-term offspring health.

## 2. Materials and Methods

### 2.1. Study Design

This study was designed as a prospective observational cohort study to investigate the relationship between maternal systemic inflammation during late pregnancy and fetal telomere length at birth. The research was conducted over a period of 24 months (January 2022–February 2023) at the Obstetrics and Gynecology Clinic of the “Pius Brînzeu” Emergency County Hospital, a tertiary care maternity facility affiliated with the Victor Babeș University of Medicine and Pharmacy in Timișoara, Romania.

The hospital serves as a regional referral center, managing both low-risk and high-risk pregnancies from across western Romania.

The study design allowed for the prospective enrollment of eligible pregnant women, with standardized biospecimen collection (maternal and umbilical cord blood) and subsequent analysis of inflammatory markers and fetal telomere length.

To further leverage the strengths of this prospective framework, maternal blood was systematically sampled within a fixed gestational window (34–36 weeks) before any labor-related immunological shifts could occur. This temporal standardization, coupled with real-time follow-up until delivery, minimizes reverse causality concerns and enhances comparability across participants. Furthermore, we prospectively designated the IL-6/IL-10 ratio as our primary inflammatory exposure, reasoning that it captures the net balance between NF-κB-driven pro-inflammatory activity and STAT3-mediated anti-inflammatory responses, thus providing a mechanistically meaningful predictor of telomere maintenance beyond individual cytokine levels.

Before initiation, the study received full ethical approval from the Institutional Ethics Committee of the “Victor Babeș” University of Medicine and Pharmacy, Timișoara (Approval number: 265/22 September 2021). All study procedures complied with the principles outlined in the Declaration of Helsinki and the relevant national legislation regarding biomedical research involving human participants. Written informed consent was obtained from each participant after a detailed explanation of the study objectives, procedures, potential risks, and confidentiality measures.

To ensure methodological consistency, trained obstetrics residents and laboratory staff under the supervision of the principal investigator collected data and processed samples.

### 2.2. Participants

Eligible participants were recruited from among pregnant women attending routine antenatal care at the Obstetrics and Gynecology Clinic of the “Pius Brînzeu” Emergency County Hospital in Timișoara, Romania. Recruitment occurred during the third trimester (gestational weeks 32 to 36), in the context of scheduled outpatient visits or during pre-delivery hospital admission.

**Figure 1:**
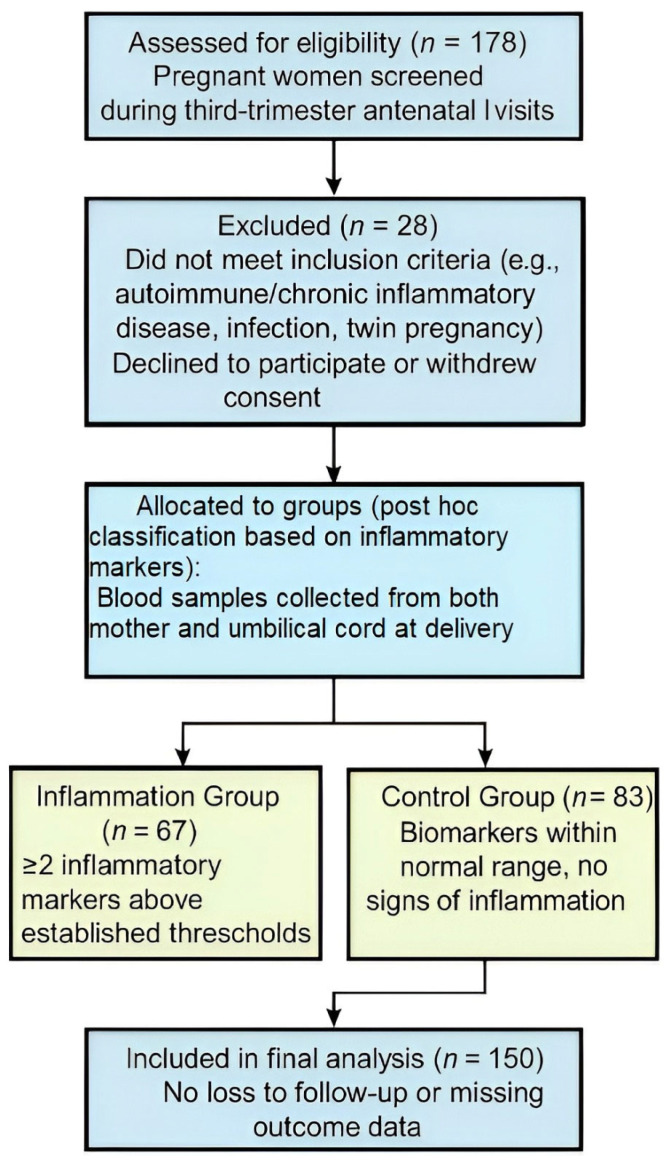
Participant flow diagram for cohort inclusion and analysis.

Out of 178 pregnant women initially assessed for eligibility, 28 were excluded—17 due to medical or pregnancy-related criteria and 11 who declined participation. A total of 150 women were enrolled and gave informed consent (Figure 1). Based on their inflammatory profiles, they were then post hoc allocated into two groups: 67 in the inflammation group and 83 in the control group. All 150 participants were successfully included in the final analysis, with no losses or missing data. Image 1 offers a concise visual summary of the study’s recruitment, grouping, and analytical structure.

Participants were eligible for inclusion if they were pregnant women aged 18 years or older, carrying a singleton pregnancy confirmed by first-trimester ultrasound, with a gestational age between 32 and 36 weeks at the time of enrollment. Additional requirements included the ability to provide informed written consent, willingness to participate in the study procedures, and agreement to provide both maternal peripheral blood and umbilical cord blood samples for biomarker and telomere length analyses. To ensure uniform perinatal conditions, all mothers in our cohort underwent elective Caesarean delivery before labor onset for obstetric indications unrelated to telomere biology, namely previous Caesarean scar, suspected cephalopelvic disproportion, persistent malpresentation, placenta previa/accreta, obstructive fibroids, prior full-thickness uterine surgery, pelvic deformity, or congenital uterine anomaly with non-vertex lie.

Women were excluded if they had a known diagnosis of chronic inflammatory or autoimmune diseases (e.g., systemic lupus erythematosus, rheumatoid arthritis), active infections (including HIV, hepatitis B/C), or were receiving immunosuppressive, corticosteroid, or anti-inflammatory therapies within 30 days before sample collection. Other exclusion criteria included acute febrile illness at enrollment, multifetal pregnancies, major fetal anomalies, intrauterine growth restriction, preterm premature rupture of membranes, and any maternal or fetal condition known to alter inflammatory status or telomere dynamics significantly.

All participants underwent a standardized screening process, including a medical interview, review of antenatal records, and routine obstetric ultrasound. Sociodemographic and clinical data—including maternal age, parity, pre-pregnancy body mass index (BMI), smoking status, educational level, and medical history—were collected using a structured questionnaire administered by trained study staff.

Women who met the eligibility criteria and consented to participate were enrolled into the study and assigned a unique anonymized study ID. Maternal venous blood was collected at the time of enrollment, and umbilical cord blood was obtained immediately after delivery. All biospecimens were labeled, processed, and stored according to predefined standard operating procedures to ensure the consistency and reproducibility of downstream analyses.

Two independent researchers extracted standardized delivery records containing neonatal data, including birth weight, gestational age at birth, sex, and Apgar scores, to ensure accuracy. The study aimed to enroll a minimum of 100 mother–infant dyads, accounting for potential exclusions due to sample quality, withdrawal of consent, or incomplete data.

### 2.3. Sample Collection and Plasma Isolation

In addition to inflammatory markers and telomere measurements, maternal and neonatal covariates were collected based on their known relevance to both inflammation and cellular aging. Maternal variables included age, pre-pregnancy BMI, parity, active smoking status, level of education, and the presence of any pregnancy-related complications such as gestational diabetes or hypertensive disorders. Neonatal data comprised gestational age at birth, birth weight, sex, mode of delivery, and Apgar scores at 1 and 5 min. Clinical and obstetric data were extracted from electronic medical records, while sociodemographic information was obtained through structured questionnaires completed at enrollment. All data entries were verified independently by two researchers to ensure completeness and accuracy.

Maternal venous blood (EDTA, 8 mL) was drawn between 34 and 36 weeks, kept on ice, and centrifuged within 30 min at 1500× *g* for 10 min at 4 °C. Plasma was aliquoted (500 µL) into polypropylene cryovials, flash-frozen in liquid nitrogen, and stored at −80 °C (single freeze–thaw allowed). Umbilical cord blood (2 mL EDTA) was collected immediately after delivery; buffy-coat leucocytes were isolated for DNA extraction using the Maxwell^®^ RSC Buffy Coat DNA Kit and processed on a Maxwell^®^ RSC 48 Instrument (Promega Corporation, Madison, WI, USA).

### 2.4. Inflammatory Marker Analysis (ELISA)

High-sensitivity C-reactive protein (hsCRP), interleukin-6 (IL-6), tumor necrosis factor-α (TNF-α), and interleukin-10 (IL-10) were quantified in duplicate with commercial ELISA kits (R&D Systems, Minneapolis, MN, USA; cat. nos. DCRP00, HS600B, DTA00D, D1000B, respectively). Plasma was diluted 1:2 for cytokines (neat for hsCRP). Plates were read at 450 nm with 570 nm reference on a FLUOstar OPTIMA microplate reader. Seven-point standard curves (4-parameter logistic fit, R^2^ > 0.99) spanned 0.156–10 pg mL^−1^ for cytokines and 0.1–10 mg L^−1^ for hsCRP.

### 2.5. Quality Control Procedure

Low- and high-control sera (Lyphochek^®^, Bio-Rad, Skokie, IL, USA) were included on every plate. Run acceptance criteria were intra-assay CV < 10% and inter-assay CV < 15%. Ten per cent of samples were re-assayed by a blinded technician; mean difference was <5%. Quarterly spike-and-recovery (acceptable 80–120%) and linearity-of-dilution (90–110%) checks verified assay performance.

### 2.6. Telomere Length Assay

Peripheral venous blood (2 mL, EDTA) was centrifuged immediately after collection; buffy-coat leukocytes were harvested and genomic DNA extracted with the Maxwell^®^ RSC Buffy Coat DNA Kit on a Maxwell^®^ RSC 48 Instrument (Promega, Madison WI, USA) per the manufacturer’s protocol. DNA purity (A_260_/A_280_ ≥ 1.8) was verified with a NanoDrop One.

Absolute telomere length was measured by monochrome multiplex qPCR following Cawthon 2002 (Nucleic Acids Res 30:e47) [19], using the Absolute Human Telomere Length Quantification Kit (ScienCell, Carlsbad, CA, USA) on an ABI 7900HT system (Thermo Fisher Scientific, Waltham, MA, USA). Each 20 µL reaction contained 5 ng genomic DNA, 10 µL SYBR Green master mix, 1 µL telomere-specific primer mix, 1 µL single-copy gene (SCG; 100 bp region on chromosome 17) primer mix, and nuclease-free water. Thermal profile: 95 °C × 10 min, then 40 cycles of 95 °C × 15 s and 60 °C × 60 s, followed by a 60–95 °C melt curve. Fluorescence derives exclusively from SYBR Green intercalation; no fluorescently labeled primers are employed.

Samples, a calibrator DNA of known telomere length, and no-template controls were run in triplicate. Replicates with SD > 0.25 Cq were repeated; a pooled inter-plate control limited inter-run CV to <7%. Telomere (T) and SCG (S) Cq values yielded ΔCq = Cq_T–Cq_S, and ΔΔCq relative to the calibrator. The kit standard curve converts the ΔΔCq-derived T/S ratio to total telomeric length (TTL) in megabases per diploid cell (Mb cell^−1^). For additional interpretability, TTL values were also expressed as kilobases per telomere (kb/tel) by dividing the Mb cell^−1^ estimate by the 92 telomeres in a human diploid genome and multiplying by 1000.

In the present study, fetal telomere length was measured from peripheral blood mononuclear cells isolated from umbilical cord blood collected immediately after delivery. PBMCs were obtained by harvesting the buffy coat from EDTA-treated cord blood samples, followed by genomic DNA extraction. The use of PBMCs as analytic target cells for telomere length assessment is well established, as they provide a readily accessible and biologically relevant source of high-quality genomic DN [18,20,21].

### 2.7. Statistical Analysis

All statistical analyses were performed using GraphPad Prism version 7.04 (GraphPad Software, La Jolla, CA, USA). Descriptive statistics were expressed as means ± standard deviations for normally distributed variables, and as medians with interquartile ranges for non-normally distributed data. The normality of data distribution was assessed using the Shapiro–Wilk test.

Comparisons between the inflammation and control groups were conducted using the independent samples *t*-test for normally distributed continuous variables and the Mann–Whitney U test for non-normally distributed variables. Categorical variables were compared using the Chi-square test or Fisher’s exact test, as appropriate.

Correlations between maternal inflammatory markers and fetal telomere length were assessed using Pearson’s correlation coefficient for normally distributed variables and Spearman’s rank correlation coefficient for skewed data. To evaluate independent predictors of fetal telomere length, a multivariable linear regression model was constructed, including relevant inflammatory markers and maternal covariates identified a priori. A two-tailed *p*-value of < 0.05 was considered statistically significant.

The sample size was determined based on data availability and recruitment feasibility within the study period, but it was also retrospectively assessed for statistical power. Assuming a medium effect size (Cohen’s d = 0.5) for differences in fetal telomere length between groups, the sample of 150 participants provided over 80% power to detect significant group differences at an alpha level of 0.05. This sample size was also sufficient to perform multivariable regression analyses, adjusting for relevant maternal and neonatal covariates.

## 3. Results

Table 1 reveals a striking similarity between the two groups regarding maternal sociodemographic and clinical characteristics. No statistically significant differences were identified, indicating that maternal age, pre-pregnancy BMI, parity, smoking status, educational attainment, employment, marital status, and urban or rural residence are evenly distributed between women with and without inflammatory status.

This level of homogeneity is particularly important from a methodological perspective, as it reduces the likelihood that baseline differences confound any observed outcomes. In other words, inflammation does not appear to be associated with distinct sociodemographic or lifestyle patterns.

Moreover, the consistency across behavioral factors (such as smoking during pregnancy and employment status) further supports the notion that other, less overt determinants may influence inflammatory status. Overall, the comparable profiles across groups strengthen the study’s internal validity and provide a solid foundation for the interpretation of subsequent analyses.

**Figure 2:**
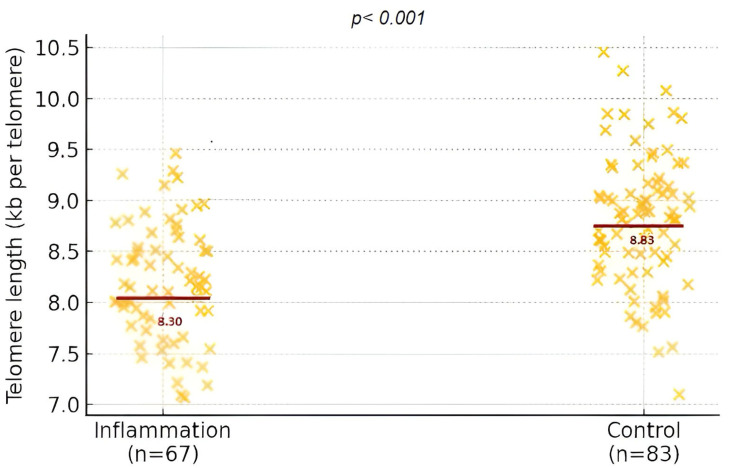
Distribution of fetal telomere length expressed in kilobases per telomere (kb/tel) in the inflammation and control groups.

The inflammatory and fetal cellular markers (Figure 2) presented in Table 2 evidence a distinct biological contrast between the two maternal groups. The findings highlight a clear biological divergence between the groups, with consistently elevated pro-inflammatory markers observed among mothers in the inflammation group. The levels of hsCRP, IL-6, and TNF-α were significantly higher, reflecting a heightened systemic inflammatory response. In contrast, IL-10 contributes to a pronounced imbalance in the immune profile, as underscored by the elevated IL-6/IL-10 ratio.

What stands out is the statistical significance of these differences and their biological relevance. The altered inflammatory environment in the maternal system appears to extend its influence to the fetus: telomere length, a proxy for cellular aging and long-term health potential, was significantly shorter in the offspring of mothers with inflammatory profiles. Maternal telomere length did not differ between groups (6.23 ± 2.92 kb vs. 6.35 ± 3.30 kb; *p* = 0.816).

Together, these results suggest a meaningful association between maternal inflammation and fetal cellular integrity.

A compelling inverse relationship emerges between maternal inflammation and fetal cellular aging, indicating that heightened inflammatory activity in the mother may be linked to accelerated telomere shortening in the developing fetus (Table 3). Elevated levels of pro-inflammatory markers—including IL-6, TNF-α, and hsCRP—were significantly associated with shorter telomeres in the fetus, suggesting that a more pronounced inflammatory state in the mother may negatively influence telomere integrity during fetal development.

Interestingly, the anti-inflammatory cytokine IL-10 showed a modest but positive correlation with telomere length, reinforcing the notion that a balanced immune environment may offer a protective effect (Table 4). The strongest association was observed with the IL-6/IL-10 ratio, highlighting the importance of individual markers and the inflammatory balance between opposing immune pathways.

These findings suggest that maternal immune activity, particularly the interplay between pro- and anti-inflammatory signals, may have direct molecular implications for the fetus, potentially shaping biological aging processes before birth.

Multivariable regression analysis underscores the independent contribution of maternal inflammation to fetal telomere length, even after adjusting for common maternal and pregnancy-related factors. Among the predictors, IL-6 and hsCRP emerge as significant negative determinants, suggesting that systemic inflammation in the mother plays a measurable role in shaping the biological aging process of the fetus.

Although IL-10 shows a positive trend, its borderline significance hints at a potential protective effect that may not be fully captured in this model. In contrast, variables such as pre-pregnancy BMI, smoking, maternal age, and gestational age at delivery do not appear to exert a strong or statistically significant influence when considered alongside inflammatory markers.

These findings highlight inflammation, not general maternal characteristics, as a key biological driver of fetal telomere dynamics, reinforcing the idea that intrauterine immune signaling may have lasting effects on the molecular trajectory of the developing child.

## 4. Discussion

This study adds to growing evidence that even low-grade maternal inflammation during pregnancy can have meaningful effects on fetal biology, particularly on telomere length at birth. The association between maternal inflammatory status and shorter fetal telomeres supports the idea that the intrauterine environment not only influences fetal growth but may also shape long-term biological trajectories at the molecular level [22,23].

The choice of peripheral blood mononuclear cells from cord blood as the target cell population for telomere length measurement is particularly relevant in the context of offspring studies. PBMCs reflect systemic immune status, are sensitive to prenatal environmental and inflammatory exposures, and have been consistently used as a surrogate for assessing cellular aging processes in early life. Moreover, telomere length in cord blood PBMCs has been shown to track into later life, making it a robust biomarker for investigating early-life programming and long-term disease risk [18,20,21].

Previous studies have reported similar findings, linking higher maternal levels of CRP and IL-6 with reduced telomere length in cord blood [18]. Research published in *JAMA Pediatrics* and the *American Journal of Obstetrics and Gynecology* has also highlighted the role of prenatal inflammation in epigenetic alterations and increased oxidative stress, both of which are known to affect telomere dynamics [24]. What makes the present findings particularly valuable is the integrated approach: instead of focusing on a single marker, the analysis covered both pro- and anti-inflammatory cytokines in a clinically healthy pregnancy population, providing a broader view of immune balance and fetal outcomes.

A key point is the consistent inverse relationship between pro-inflammatory markers, especially IL-6 and hsCRP, and fetal telomere length. IL-6 drives inflammation and oxidative stress by activating intracellular pathways like JAK/STAT, which promote reactive oxygen species and DNA damage. hsCRP, while less specific, is a well-established marker of systemic inflammation, and its elevated levels, even in pregnancies without infection, may indicate chronic low-grade immune activation [25,26,27]. Together, these markers suggest that a more inflammatory maternal state could translate into increased fetal biological stress.

Our findings extend and refine the current understanding of prenatal inflammation and telomere biology in three key ways. Firstly, by modeling the IL-6/IL-10 ratio alongside individual cytokines and hsCRP, we show that immune balance—rather than absolute pro-inflammatory load—explains the largest share of fetal telomere variability (≈28%), doubling the variance captured by IL-6 alone.

Secondly, in contrast with the seminal prospective study of Lazarides et al. 2019 [15], which quantified only IL-6 and TNF-α across a broad 24–40-week gestational window and included pregnancies with metabolic and psychosocial comorbidities, our cohort was clinically uncomplicated, sampled in a narrow 34–36-week window, and analyzed with a pre-specified multimarker framework that integrates an anti-inflammatory axis.

Thirdly, multivariable adjustment revealed that inflammatory signatures, not maternal demographics or obstetric factors, independently predict fetal telomere shortening, underscoring systemic immune status as a modifiable driver of early-life biological aging. Together, these advances shift the field from single-marker associations to a mechanistic, clinically actionable paradigm centered on immune balance, opening avenues for targeted interventions (e.g., nutritional ω-3, stress reduction) aimed at preserving telomere length before birth.

IL-10, on the other hand, showed a weak but positive association with fetal telomere length. Although this correlation narrowly missed conventional significance, the trend aligns with the idea that IL-10 may have a protective effect [15]. As a key anti-inflammatory cytokine, IL-10 limits the production of IL-6 and TNF-α and helps maintain immune homeostasis [28,29,30]. Importantly, the IL 6/IL 10 ratio emerged as one of the strongest indicators of telomere attrition, highlighting that it is not just about the absolute levels of cytokines but also about the balance between inflammatory and anti-inflammatory forces. For instance, elite athletes with concomitantly elevated IL 6 and IL 10 had longer telomeres than peers with lower sport intensity, indicating that a balanced cytokine milieu supports telomere maintenance [31]. Moreover, population-level evidence suggests that telomere shortening associates with elevated IL 6, while IL 10 increases concomitantly as a compensatory anti-inflammatory response, further emphasizing that a balanced IL 6/IL 10 ratio—not just single-cytokine levels—may be critical in modulating telomere attrition [32].

What further strengthens these findings is that the association between maternal inflammation and fetal telomere length remained significant even after adjusting for maternal age, BMI, smoking status, and gestational age at birth. These are well-known factors that could potentially influence telomere biology, yet they did not explain the observed variation. This reinforces the notion that maternal immune status functions as an independent biological driver of fetal telomere dynamics [15]. Similarly, in the PM2.5 study, associations with shorter cord blood telomeres remained significant after adjustment for maternal age, BMI, smoking, education, gestational age, birth complications, and more, highlighting that maternal immune/inflammatory exposures independently shape fetal telomere dynamics [33].

From a physiological perspective, there are several mechanisms that might explain these effects. Inflammatory cytokines and immune mediators can cross or affect the placenta, leading to increased oxidative stress and DNA damage within fetal tissues. Additionally, inflammation may disrupt placental antioxidant capacity, exposing the fetus to a pro-oxidative intrauterine environment. This has the potential to impair telomerase function and accelerate telomere shortening, especially in rapidly dividing fetal cells [17,26,34,35].

Clinically, these findings carry important implications. Telomere length at birth is increasingly seen as a marker of early biological aging and a predictor of later susceptibility to chronic diseases, including cardiovascular conditions, metabolic syndrome, and even certain cancers. Even subtle changes in maternal immune status during pregnancy—changes that might go unnoticed in routine care—could shape long-term health outcomes for the child. Newborn telomere length measured in cord blood predicts telomere length throughout life and correlates with midlife cardiovascular risk, reinforcing its role as a foundational biomarker for lifelong health trajectories [6,36].

These insights suggest that monitoring inflammatory markers in pregnancy could become a valuable tool in obstetric care, especially for women at higher risk due to obesity, metabolic imbalance, or psychosocial stress. Early identification of elevated inflammation could lead to interventions, whether through nutrition, stress reduction, or other personalized strategies, to promote a more favorable intrauterine environment.

These results support the idea that pregnancy represents a critical window for programming health across the lifespan [15,37]. Even when not clinically apparent, maternal inflammation seems to play a central role in shaping the child’s molecular foundation [15,38]. Understanding and addressing this could offer new ways to optimize health before birth even occurs.

Given the growing recognition of telomere length as a marker of early-life programming, future research should explore whether maternal inflammation-induced telomere shortening at birth translates into measurable health outcomes later in childhood or adulthood. Longitudinal studies tracking telomere dynamics over time, alongside neurodevelopmental, metabolic, and cardiovascular markers, could offer deeper insights into the lasting impact of the intrauterine inflammatory environment. It would also be valuable to investigate whether targeted interventions, such as anti-inflammatory dietary patterns, stress reduction programs, or microbiome modulation, can mitigate inflammatory effects during pregnancy and preserve telomere integrity in the fetus. Additionally, integrating other layers of molecular data, including epigenetic changes and mitochondrial function, could help build a more complete picture of how maternal immune activity influences fetal cellular aging.

Maternal inflammation may be particularly consequential in the context of gestational surrogacy, where the genetic parents and the gestational carrier differ. Our data suggest that low-grade systemic inflammation during late pregnancy shortens fetal telomere length at birth; given that telomere dynamics are largely established in utero and track with later-life health risks, any inflammatory milieu experienced by a surrogate could impart lasting biological effects on the child, independent of the parents’ genome. These observations underscore the need to monitor and, where possible, mitigate inflammatory conditions in surrogate pregnancies, both to optimize neonatal outcomes and to inform ethical guidelines and clinical counseling for intended parents and gestational carriers alike.

### Strengths and Limitations

One of the main strengths of this study lies in its prospective design and well-defined cohort of clinically healthy pregnancies, which allowed for standardized biospecimen collection and minimized potential confounders related to overt maternal or fetal pathology. The simultaneous measurement of multiple inflammatory markers—both pro- and anti-inflammatory—offers a more nuanced understanding of the maternal immune environment and its impact on fetal telomere biology. Additionally, the use of validated, high-sensitivity assays for biomarker quantification and telomere measurement adds robustness to the data quality and reliability.

Nonetheless, our prospective observational design can only suggest temporality, not causality. Future randomized or interventional studies targeting the IL-6/IL-10 axis are required to confirm a causal role.

However, some limitations should be acknowledged. Firstly, the study’s observational nature precludes any direct conclusions about causality. Although multiple confounders were controlled for, residual confounding cannot be entirely excluded. Secondly, while telomere length in cord blood reflects fetal cellular aging, it may not fully capture telomere dynamics across different tissues. Moreover, the inflammatory profile was assessed at a single time point in late pregnancy, which may not reflect fluctuations across the entire gestational period. Lastly, although the sample size was adequate for statistical power, larger multicenter studies would be valuable to enhance generalizability and explore potential subgroup effects. As with all observational studies, our design cannot establish definitive causality, and the associations observed should be interpreted as indicative rather than conclusive evidence of the underlying biological mechanisms.

Available longitudinal data indicate that telomere length remains relatively stable during the first years of life, underscoring the relevance of values measured at birth as a reference point for intrauterine exposure [21,39,40].

## 5. Conclusions

This study demonstrates that maternal immune imbalance in late pregnancy—particularly an elevated IL-6/IL-10 ratio—acts as a biologically relevant determinant of fetal telomere shortening, independent of common clinical risk factors. The inflammatory signature, rather than maternal demographics or obstetric history, emerged as the critical predictor of early cellular aging.

These findings suggest that low-grade, subclinical inflammation during pregnancy is not benign but may actively program molecular aging pathways in the fetus. In this context, inflammatory biomarkers could serve as early indicators of intrauterine biological stress, even in otherwise healthy pregnancies. Integrating immune profiling into prenatal risk assessment may enable more precise identification of fetuses at risk of accelerated cellular aging.

Furthermore, targeting maternal inflammation—through nutritional, behavioral, or pharmacological strategies—could become a feasible avenue for telomere-preserving interventions during pregnancy. This positions maternal immune status not just as a correlate, but as a modifiable factor with downstream consequences for offspring longevity and disease vulnerability.

## Figures and Tables

**Table 1:** Maternal sociodemographic and clinical characteristics by inflammatory status.

Variable	Inflammation Group (n = 67)	Control Group (n = 83)	*p*-Value
Maternal age (years)	29.8 ± 4.6	30.1 ± 4.4	0.684
Pre-pregnancy BMI (kg/m^2^)	27.3 ± 3.9	26.9 ± 3.7	0.521
Primiparous	35 (52.23%)	40 (48.19%)	0.742
Smoking during pregnancy	11 (16.41%)	12 (14.45%)	0.917
Educational level—high school or less	24 (35.82%)	27 (32.53%)	0.802
Educational level—university degree	43 (64.17%)	56 (67.46%)	0.802
Employment status—employed	49 (73.13%)	63 (75.90%)	0.842
Marital status—married/cohabiting	59 (88.05%)	75 (90.36%)	0.850
Residence—urban	41 (61.19%)	53 (63.85%)	0.868

**Table 2:** Inflammation and telomere lengths of mothers and fetuses, by group.

Variable	Inflammation Group (n = 67)	Control Group (n = 83)	*p*-Value
hsCRP (mg/L)	6.4 ± 2.2	2.3 ± 1.4	<0.001
IL-6 (pg/mL)	4.7 ± 1.5	2.2 ± 1.0	<0.001
TNF-α (pg/mL)	3.8 ± 1.1	2.6 ± 0.9	<0.001
IL-10 (pg/mL)	1.8 ± 0.6	2.5 ± 0.7	<0.001
IL-6/IL-10 ratio	2.1 ± 0.5	1.3 ± 0.4	<0.001
Maternal telomere length	6.23 ± 2.92 kb	6.35 ± 3.30 kb	0.816
Fetal telomere length	8.30 ± 0.63 kb	8.83 ± 0.66 kb	<0.001

**Table 3:** Correlations between maternal inflammatory markers and fetal telomere length (kb per telomere).

Variable	Correlation with Fetal Telomere Length (r)	*p*-Value	Test Used
hsCRP (mg/L)	−0.42	<0.001	Spearman
IL-6 (pg/mL)	−0.49	<0.001	Pearson
TNF-α (pg/mL)	−0.38	0.002	Pearson
IL-10 (pg/mL)	+0.25	0.031	Pearson
IL-6/IL-10 ratio	−0.53	<0.001	Pearson

**Table 4:** Multivariable linear regression analysis predicting fetal telomere length (kb per telomere).

Predictor Variable	Standardized β	95% CI	*p*-Value
IL-6 (pg/mL)	−0.37	−0.51 to −0.22	<0.001
hsCRP (mg/L)	−0.29	−0.43 to −0.11	0.004
IL-10 (pg/mL)	+0.18	−0.01 to 0.38	0.054
Pre-pregnancy BMI (kg/m^2^)	−0.12	−0.27 to 0.02	0.098
Smoking status (yes vs. no)	−0.09	−0.24 to 0.05	0.191
Maternal age (years)	−0.06	−0.20 to 0.08	0.377
Gestational age at delivery (weeks)	+0.04	−0.10 to 0.17	0.565

## Data Availability

The data are available from the corresponding author of the study. You may contact the corresponding author for further details and access to the relevant data. Additionally, a copy of the data is also stored in our clinic’s records.
